# ROC-1, P21 and CAIX as markers of tumor aggressiveness in bladder carcinoma in Egyptian patients

**DOI:** 10.1186/s13000-020-00947-7

**Published:** 2020-04-07

**Authors:** Dalia Rifaat Al-Sharaky, Mona Abd El-Halim Kandil, Hayam Abdel Samie Aiad, Enas Megahed El-hosary, Hagar Abdelmagied Alagizy, Mahmoud Abdel-Sattar Elshenawy, Hala Said El-Rebey

**Affiliations:** 1 Department of Pathology, Faculty of Medicine, Menoufia University, Shebin El-Kom, Menoufia Governorate 35211 Egypt; 2 General hospital, Shebin El-Kom, Menoufia Governorate, Egypt; 3 Clinical oncology & Nuclear medicine Department, Faculty of Medicine, Menoufia University, Shebin El-Kom, Egypt

**Keywords:** ROC-1, CAIX, P21, BC, Immunohistochemistry, Prognosis

## Abstract

**Background:**

Bladder cancer (BC) is one of the most common malignancies in Egypt, representing about 8.7% of cancers in both sexes with more predominance in males, making identification of valuable predictive and prognostic markers, mandatory. Cullin-RING ligases (CRL) play an important role in the ubiquitination of cell cycle-related proteins or other proteins (e.g., DNA replication protein, signal transduction protein). Regulator of Cullins-1 (ROC-1) is a key subunit of CRL. P21 belongs to the family of cyclin dependent kinase inhibitors (CKIs) which regulates cell cycle by inactivating Cyclin- Dependent Kinases key regulators of the cell cycle. CAIX a highly active member of the family of carbonic anhydrases has gained much interest as a hypoxic marker. Hypoxia is a consequence of the rapid growth of many tumors, including bladder cancer, and is an important regulator of gene expression and resistance to chemotherapy and radiotherapy. Therefore the purpose of this study is to evaluate the role of ROC-1, CAIX and P21 and its relationship with the clinico-pathological features of bladder cancer in Egyptian patients.

**Methods:**

Using the standard immunohistochemical technique, ROC-1, CAIX and P21 expression in 80 primary bladder carcinomas and 15 normal bladder specimens as control group were assessed. The bladder carcinoma cases included 50 cases with muscle invasive bladder cancer and 30 cases with non-muscle invasive bladder cancer.

**Results:**

Over expression of ROC-1, CAIX and P21 in BC were significantly associated with muscularis propria invasion and high grade BC. ROC-1, CAIX and P21, showed significant inverse relationship in primary BC cases. CAIX expression was significantly higher in BC compared with controls. Regarding the survival analysis, expression of ROC-1, CAIX and P21 didn’t affect the survival of BC patients.

**Conclusions:**

High expression of ROC-1, CAIX and P21 could be promising potential biomarkers for identifying patients with poor prognostic factors in bladder cancer serving as potential targets for cancer therapy.

## Background

In Egypt bladder cancer is the third common malignancy accounting for 8.7% of all cancers according to National Cancer Registry with expected new cases about 10,709 by 2020, 12,762 by 2025 and 28, 337 by 2050 [1, 2]. Interest in understanding the molecular profiling of bladder carcinoma, to understand the biology of these tumors and to develop novel therapies has developed.

Cullin-RING ligases (CRL) are the largest family of E3 ubiquitin ligases [3]. Regulator of Cullins-1 (ROC-1) is a key subunit of CRL, also known as RING box protein-1 (RBX1) [4]. ROC-1 knockdown arrest the cells at G2/M phase of cell cycle by up regulating cyclin B1, P21 and P27 which is a useful strategy in treating various human cancers [5]. Some studies proved that ROC-1 protein is overexpressed in non-muscle invasive bladder cancer, suggesting its potential role in bladder cancer development and progression [6].

P21 belongs to the CIP/KIP (CDK Interacting Protein/Kinase Inhibitor Protein) family of cyclin dependent kinase inhibitors (CKIs) that includes also p27 and p57. These proteins were identified for their ability to regulate cell cycle by inactivating Cyclin- Dependent Kinases (CDKs), key regulators of the cell cycle. P21 has a different prognostic role in several cancers and according to the cellular context in which it is expressed; it can have a dual role being a target of specific therapies, or a marker of poor prognosis [7].

CAIX is a highly active member of the family of carbonic anhydrases with an ability to catalyze efficiently the reversible hydration of carbon dioxide to carbonic acid. CAIX can be designated as cancer-related protein which is almost exclusively associated with tumors, and is overexpressed in some tumors. Therefore, inhibition of CAIX is considered as a promising therapeutic target for the treatment of solid tumors where hypoxic environment has developed [8].

The aim of this study is to evaluate the immunohistochemical expression of ROC-1, CAIX and p21 in correlation with the clinicopathological and prognostic parameters.

## Methods

This retrospective study included 80 primary bladder carcinoma and 15 normal bladder specimens (control group). The bladder carcinoma cases included 50 cases with MIBC and 30 cases of NMIBC. The cases were retrieved from the archives of Pathology Department, Faculty of Medicine, Menoufia University spanning the period between January 2014 and December 2016.

### Clinical data of the studied groups

Clinical data were obtained from patients’ medical records and documented in (Table 1).

### Histopathological assessment

The hematoxylin and eosin (H&E) stained sections were evaluated for the followings; Histological type according to WHO classification, 2016. Tumor grading was done according to WHO/ISUP grading criteria [9]. The mitosis and apoptosis were counted semi quantitatively in ten randomly selected high power fields [10]. Depth of invasion and staging of the tumor were defined according to TNM American Joint Committee on Cancer-Union International Center Cancer staging system (AJCC-UICC) which classifies the tumor histologically as NMIBC (stage pTa and pT1) or MIBC (stage pT2, pT3 and pT4) [9].

### Immunohistochemistry

The method used for immunostaining was streptavidin-biotin amplified system. Sections cut from the paraffin-embedded blocks were stained with Anti-ROC-1 (cat# SC-5200, Santa Cruz) purified mouse monoclonal antibody received as 0.1 ml conc. and diluted by phosphate buffer saline (PBS) in a dilution of 1:100. Anti-P21 (cat# YPA1643, Snuff) rabbit polyclonal antibody received as 0.1 ml conc. and diluted by PBS in a dilution of 1:400 and Anti-CAIX (cat # YPA1250, Snuff) rabbit polyclonal antibody received as 0.1 ml conc. and diluted by PBS in a dilution of 1:100.

Tissue sections prepared from seminiferous tubules of testis, papillary thyroid carcinoma and normal gastric mucosa were used as positive control for ROC-1, P21 and CAIX respectively. Negative control slides were also included in each run by omitting the primary antibody.

Positive cases for both ROC-1and P21were assigned as long as cytoplasmic or nucleo-cytoplasmic expression in ≥10% of cells was identified [11, 12]. Cytoplasmic or nucleo-cytoplasmic staining of CAIX in > 1% of cells was defined as positive immunoreaction [13].

### Statistical analysis

The statistical analysis was conducted using SPSS “statistical package for the social science” program for windows, version 22.0 (SPSS INC., Chicago, Illinosis, USA). Contingency tables were analyzed with descriptive statistics [Arithmetic mean ($\overline{\mathrm{x}}$),Standard deviation (SD), Percentage (%), Median and Range] and analytic statistics [Chi- square test (× ^2^- test), Mann-Whitney Z test (Z test), Kruskal-Wallis test (K test), Fisher’s exact (FE)]. Overall survival (OS) was analyzed using the Kaplan–Meier method, and differences were examined using log-rank tests. Cox’s proportional hazard regression test was used to estimate univariate and multivariate hazard ratios for prognosis. *P* values of ≤0.05 were considered statistically significant [14].

## Results

### Clinicopathologic characteristics:

Clinicopathologic characteristics of primary bladder carcinoma cases are summarized in **(**Table 1).

**Table 1:** Clinicopathological data of the studied bladder carcinoma cases

Variables	Category	No. (%)
**Age**	Mean ± SD	62 ± 8.76
	Range	45–96
**Sex**	Male	72 (90%)
	Female	8 (10%)
	M:F	8: 1
**Size**	Mean ± SD	4.64 ± 2
	Range	1–10
**Muscularis propria invasion** **Urothelial carcinoma**	Non-invasive	30 (37.50%)
	Invasive	50 (62.50%)
**Histologic Type**	Infiltrating UC	58 (72.5%)
	UC with divergent differentiation (squamous or glandular)	14 (17.5%)
	Squamous cell carcinoma	5 (6.25%)
	Small cell neuroendocrine	3 (3.75%)
**Gradingofurothelial carcinoma**	Low	14 (18.7%)
	High	61 (81.3%)
**Grading of squamous cell carcinoma**	Moderately differentiated	4 (80%)
	Poorly differentiated	1 (20%)
**Bilharziasis**	Present	26 (32.50%)
	Absent	54 (67.5%)
**Necrosis**	Present	20 (25%)
	Absent	60 (75%)
**Lymph-vascular invasion**	Present	14 (17.50%)
	Absent	66 (82.50%)
**Perineural invasion**	Present	8 (10.00%)
	Absent	72 (90.00%)
**Stromal reaction**	Desmoplastic	60 (75.00%)
	Inflammatory	20 (25.00%)
**Lymph node stage (TNM)**	N0	25 (50.00%)
	N1	9 (18.00%)
	N2	16 (32.00%)
**Mitotic index**	low index	50 (62.25%)
	High index	30 (37.50%)
**Apoptotic index**	low index	44 (55.00%)
	High index	36 (45.00%)

*SD* Standard deviation, *No* number, *UC* Urothelial carcinoma

### Immunohistochemical profile of ROC-1, CAIX and p21 in the studied bladder cancer cases were summarized in (Table 2)

#### Comparison between malignant and control groups regarding ROC-1, P21 and CAIX expression (Table 3)

CAIX was significantly expressed in bladder carcinoma than in normal urothelium(*P* = 0. 01), however ROC-1 and P21 revealed no significant difference between the normal urothelium and bladder carcinoma***.***

**Table 2:** Immunohistochemical profile of the studied ROC-1,CAIX and p21 in the studied bladder carcinoma cases

Variables	Malignant cases (No = 80) No (%)
ROC1 expression	
Positive	72 (90%)
Negative	8 (10%)
	**No = 72**
RCO1 subcellular localization	
Cytoplamic	24 (33.3%)
Nucleo-cytoplasmic	48 (66.7%)
ROC-1 intensity	
Predominant strong	18 (25.0%)
Predominant moderate	30 (41.7%)
Predominant mild	24 (33.3%)
ROC1 H.score	
Low expression	37 (51.4%)
High expression	35 (48.6%)
CAX expression	
Positive	49 (92.5%)
Negative	31 (73.8%)
	**No = 49**
CAX subcellular localization	
Cytoplamic	44 (89.7%)
Nucleo-cytoplasmic	5 (10.2%)
CAIX intensity	
Predominant strong	
Predominant moderate	
Predominant mild	
CAX H-score	
Low expression	31 (63.3%)
High expression	18 (36.7%)
P21 expression	
Positive	67 (83.75%)
Negative	13 (16.25%)
	**No = 67**
P21 subcellular localization	
Cytoplamic	55 (82.1%)
Nucleo-cytoplasmic	12 (17.9%)
P21 intensity	
Predominant strong	
Predominant moderate	
Predominant mild	
P21 H-score	
Low expression	34 (50.7%)
High expression	49 (92.5%)

*No* number

**Table 3:** Comparison between ROC1, P21 and CAIX expression in control group and bladder carcinoma cases

Variables	Category	control group (***n*** = 15)	Malignant group (***n*** = 80)	Test of significance	***p***_value
**ROC1** **expression**	Negative	1 (6.66%)	8 (10%)	FE = 0.16	1
	Positive	14 (93.33%)	72 (90%)		
**ROC1 Sub cellular localization**	Cytoplasmic	8 (57.14%)	24 (33.3%)	×^2^ = 2.84	0.09
	Nucleocytoplasmic	6 (42.86%)	48 (66.7%)		
**ROC1** **H-score**	Low expression	8 (57.1%)	37 (51.4%)	X^2^ = 0.16	0.69
	High expression	6 (42.9%)	35 (48.6%)		
**P21 expression**	Negative	5 (33.3%)	13 (16.25%)	FE = 2.4	0.1
	Positive	10 (66.7%)	67 (83.75%)		
**P21 Sub cellular localization**	Cystoplasmic	8 (80%)	55 (82.1%)	FE = 0.03	1
	Nucleocytoplasmic	2 (20%)	12 (17.9%)		
**P21 H-score**	Low expression	5 (50%)	34 (50.7%)	FE = 0.002	1
	High expression	5 (50%)	33 (49.3%)		
**CAIX expression**	Negative	11 (26.2%)	31 (73.8%)	x2 = 6.13	0.01*
	Positive	4 (7.5%)	49 (92.5%)		
**CAIX Sub cellular localization**	Cystoplasmic	4 (8.3%)	44 (91.7%)	FE = 0.45	1
	Nucleocytoplasmic	0 (0%)	5 (100%)		
**CAIX H-score**	Low expression	1 (3.1%)	31 (96.9%)	FE = 0.22	1
	High expression	3 (14.3%)	18 (85.7%)		

*No* number, * significant, *FE* fisher’s exact test

### Relationship between ROC-1, P21 and CAIX expression and clinicopathological factors in primary bladder carcinoma cases

#### ROC-1

**Fig. 1:**
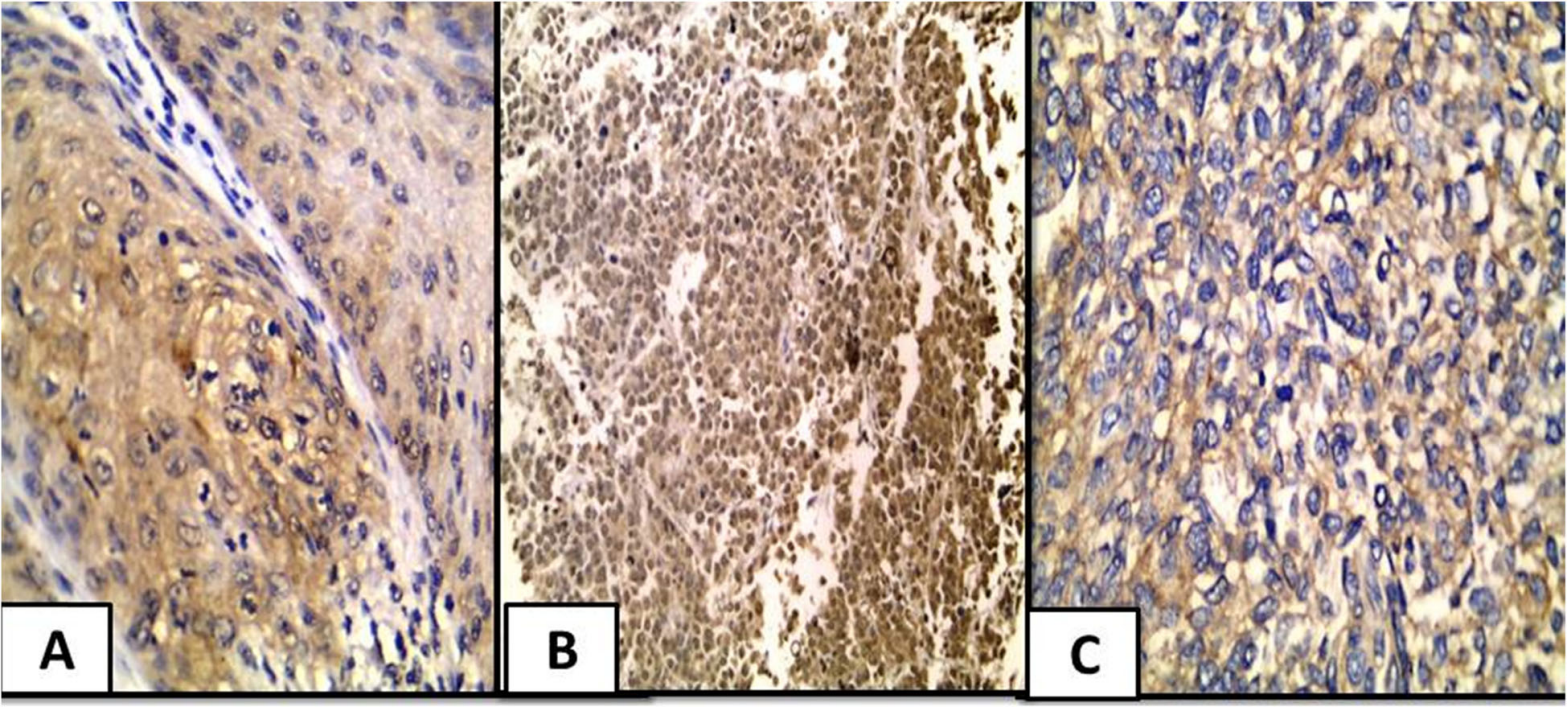
ROC-1 expression in studied cases. **a** A case of moderately differentiated squamous cell carcinoma showed positive moderate cytoplasmic expression of ROC-1 (IHC × 200/400). **b** A case of small cell neuroendocrine carcinoma showed positive strong nucleocytoplasmic expression of ROC-1 (IHC × 200) **c** A case of invasive urothelial carcinoma, high grade showed positive mild cytoplasmic expression of ROC-1 (IHC × 400)

Small sized tumors (*P* = 0.03), invasive urothelial carcinoma (*P* = 0.04), high grade UC (*P* = 0.04), cases with absent necrosis (*p* = 0.04)***,*** infiltrating UC in comparison to other types (SCC and small cell neuroendocrine carcinoma) (*P* = 0.05) and also cases that displayed desmoplastic stroma (*P* = 0.03) significantly overexpressed ROC-1. As for the subcellular localization, nucleo-cytoplasmic expression of ROC-1 was in favor of old aged patients (*P* = 0.000) and small sized tumors (*P* = 0.01) together with muscle invasive UC (*P* = 0.000), presence of bilharzial ova (*P* = 0.008) and high mitotic index (*P* = 0.004) **(**Fig. 1**).**

#### P21

**Fig. 2:**
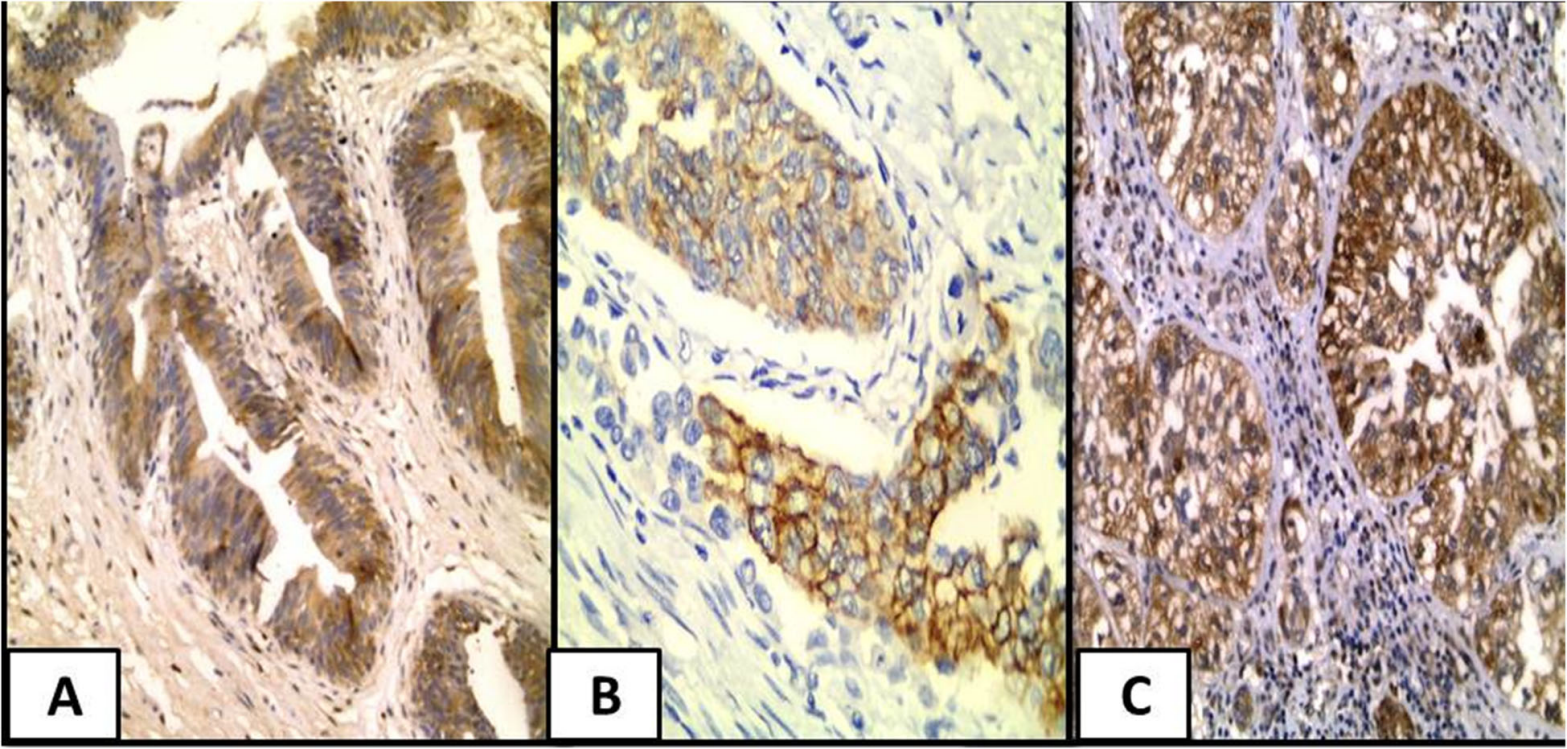
P21 expression in studied cases. **a** A case of normal urothelium showed positive P21 expression in urothelial cells (IHC × 400). **b** A case of invasive urothelial carcinoma, high grade showed positive mild cytoplasmic expression of P21 (IHC ×400). **c** A case of invasive urothelial carcinoma, high grade showed positive strong cytoplasmic expression of P21 (IHC ×400)

P21overexpression was significantly in favor of muscle invasive (*P* = 0.02), high grade UC (*P* = 0.02) and high apoptotic index (*P* = 0.05). Cytoplasmic expression of P21 was significantly in favor of muscle invasive cases (*P* = 0.001), presence of necrosis (*P* = 0.028), advanced T stage (*P* = 0.004), high apoptotic index (*P* = 0.001) and high mitotic index (*P* = 0.002) **(**Fig. 2**).**

#### CAIX

High grade urothelial carcinoma displayed significant CAIX overexpression in comparison with low grade tumors (*P* = 0.01).

**Fig. 3:**
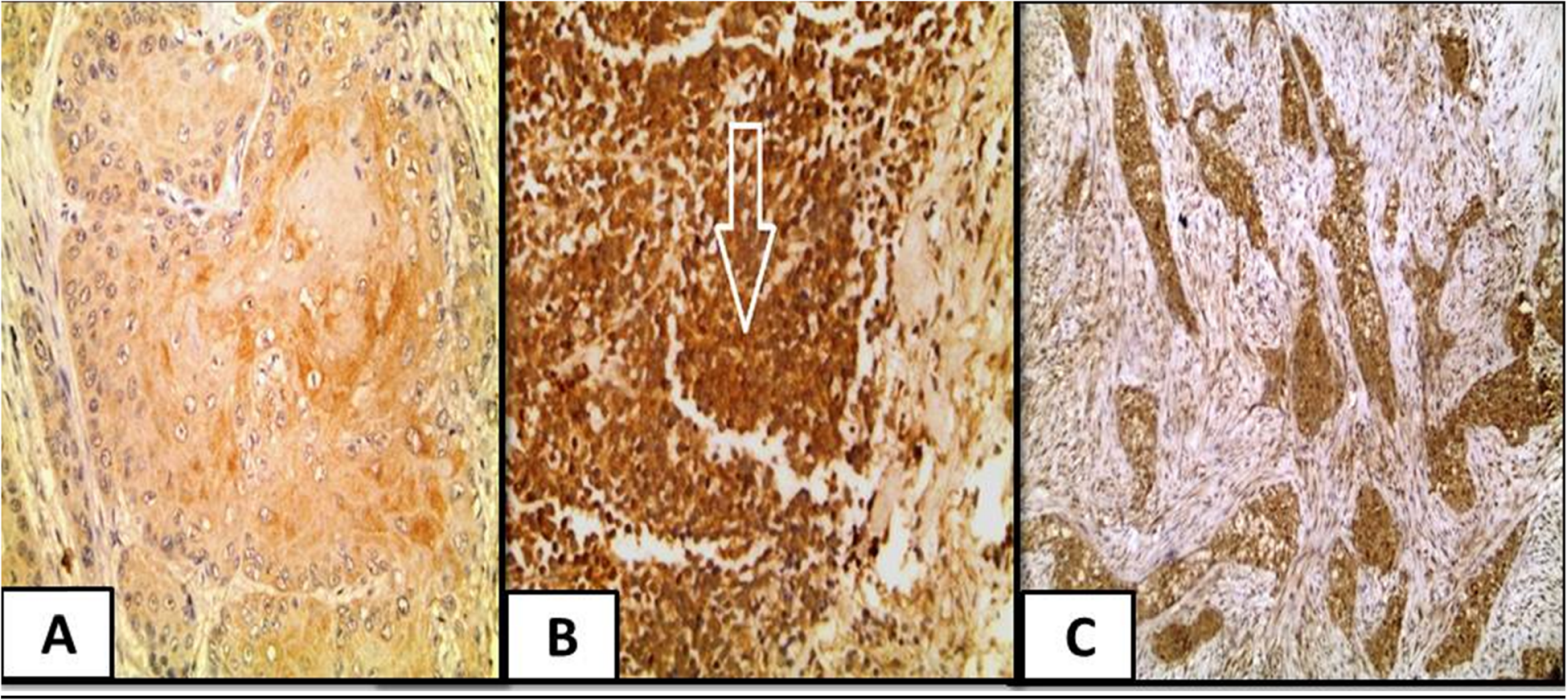
CAIX expression in studied cases. **a** A case of moderately differentiated squamous cell carcinoma showed positive moderate cytoplasmic expression of CAIX (IHC × 400). **b** A case of small cell neuroendocrine carcinoma showed positive strong cytoplasmic expression of CAIX (IHC × 400).**c** A case of invasive urothelial carcinoma, high grade with positive strong cytoplasmic expression of CAIX and positive desmoplastic stromal reaction (IHC X 200)

Furthermore, a significant association between positive CAIX expression and female gender (*P* = 0.02), presence of bilharziasis (*P* = 0.046) and desmoplastic stroma(*P* = 0.024) was present **(**Fig. 3**).**

In our study, there were 20 cases of the urothelial carcinoma that displayed necrosis; CAIX was evaluated around necrosis (perinecrotic) and away from necrotic area. In the perinecrotic area, 12 cases out of 20 displayed membranous expression of CAIX, while in areas away from necrosis 4 cases only out of 20 were positive for CAIX.

All the cases that were positive for CAIX expression in the perinecrotic area were negative in areas away from necrosis (*P* = 0.014). However, half of the cases that showed negative expression in the perinecrotic area were also negative in areas away from necrosis **(**Table 4**).**

**Table 4:** Relationship between CAIX expression in malignant cells perinecrotic and away from necrosis

Variables	Perinecrotic (No = 20)		Test of significance	*P*-Value
	Negative (No = 8)	Positive (No = 12)		
Away from necrosis (No = 20)			FE = 7.5	0.014*
Negative (no = 16)	4 (50%)	12 (100%)		
Positive (no = 4)	4 (50%)	0 (0%)		

NB: The percentage is calculated by column

* significant, *No* number, *FE* fisher’s exact test

### Correlation between ROC-1 H-score and both CAIX and P21 H-scores in malignant cases (Table 5)

There was a significant inverse relationship between ROC-1 H-score and both CAIX and P21 H-scores (*P* = 0.02 and 0.01 respectively).

**Table 5:** Correlation between ROC-1 H score and both CAIX and P21 H-scores in malignant cases

Markers	ROC 1 H-score	
	*r*	*P* value
CAIX H-score	−0.337	0.02*
P21 H-score	−0.303	0.01*

*r* Spearman’s rho, * significant

### Survival analysis

After exclusion of low grade, small cell and urothelial carcinoma with squamous differentiation, none of the studied ROC-1, CAIX or P21 expressions showed significant impact on overall survival **(**Table 6**).**

**Table 6:** Univariate survival analysis for studied bladder carcinoma cases

Variable	Overall survival (months)			
	Mean survival time	SE	Log rank	***P***-value
Gender				
Male	42.417	2.323	0.009	0.926
Female	45.429	4.508		
Age (years)				
≤ 60	43.120	2.954	0.024	0.877
> 60	42.209	2.979		
Size of mass				
Absent	44.258	3.551	0.0	0.982
Present	42.210	2.472		
Diagnosis				
TCC	42.986	2.139	0.021	0.886
Squamous ca	43.800	8.941		
Grade				
Poorly diff	30.0	0.0	1.726	0.422
Moderate differ.	47.250	10.872		
High	42.986	2.139		
Bilharsiasis				
Absent	44.632	2.654	2.271	0.132
Present	39.438	3.244		
Necrosis				
Absent	43.615	2.475	0.047	0.829
Present	40.857	3.787		
LVI				
Absent	42.828	2.354	0.066	0.798
Present	42.778	4.558		
PNI				
Absent	43.537	2.203	1.211	0.271
Present	37.833	6.263		
Stroma				
Inflammatory	41.769	3.530	0.232	0.630
Desmoplastic	43.252	2.559		
Tumor stage				
T1	44.258	3.551	1.922	0.589
T2	43.129	3.213		
T3	39.054	3.823		
T4	52.333	11.681		
Ms invaion				
Absent	44.258	3.551	0.0	0.982
Present	42.210	2.472		
Node				
Absent	41.848	2.322	0.988	0.320
Present	44.322	4.061		
Reactivity (ROC-1 Immunohistochemistry)				
Negative	59.0	0.0	1.454	0.228
Positive	42.100	2.095		
Pattern (ROC-1 Immunohistochemistry)				
Negative	59.0	0.0	2.415	0.299
Cytoplasmic	40.134	3.038		
Nuclear/cytopl	42.940	2.723		
H score (ROC-1 Immunohistochemistry)				
Low median (< 90)	43.904	2.904	0.044	0.834
High median (> 90)	41.947	2.987		
IRS (ROC-1 Immunohistochemistry)				
Low	43.519	2.454	1.323	0.250
High	40.700	3.950		
Reactivity (CAIX Immunohistochemstry				
Negative	45.948	3.149	1.077	0.299
Positive	40.856	2.703		
Pattern (CAIX Immunohistochemstry				
Negative	45.948	3.149	1.077	0.299
Cytoplasmic	40.856	2.703		
H score (CAIX Immunohistochemstry				
Low median (< 90)	43.342	3.628	3.498	0.061
High median (> 90)	36.500	3.591		

No comparison analysis was performed for urothelial carcinoma and squamous cell carcinoma grades because the factor variable had only one value

*CI* Confidence interval

## Discussion

In the present study, ROC-1 showed expression in 90 and 93% of the bladder cancer cases and normal urothelial tissues respectively. Increased expression in the BC can be explained by its role in mediating degradation of tumor suppressors and DNA replication licensing proteins which would accelerate cell growth. Furthermore, correlation between ROC-1 and Ki67 as reported by Migita and colleagues [15] might help in proliferation of cancer cells. Several studies support the increased expression of ROC-1 in BC in our study [6, 15, 16].

Overexpression of ROC-1 in the malignant group was significantly associated with poor prognostic factors in the current study as invasion into muscularis propria, and high grade UC. Increase of ROC-1 expression from early to advanced BC, supports its poor prognostic impact and implies a potential role in BC progression.

ROC-1 overexpression significant association with desmoplastic stroma was consistent with others supporting the role of ROC-1 as an EMT [17–19]. Knockdown of ROC-1 transactivates the RhoA and Rac1 signaling pathways and inhibits mTOR activity, which has a pivotal role in regulation of various cellular processes including EMT, thus causing suppression of EMT in MIBC [20–24].

The relation between ROC-1 and necrosis in BC in the present study is an inverse relation**.** This can be explained by ROC-1 mediated down regulation of hypoxic marker CAIX, for ROC-1 ubiquities VHL which degraded hypoxic marker CAIX [25–27]. So there is an inverse relation between hypoxic marker CAIX released in the hypoxic conditions as necrosis and with ROC-1.

As for the association between nucleo-cytoplasmic expression of ROC-1 and aggressive pathologic features of BC (as muscle invasion, high mitosis, presence of bilharzial infection and old age), it was supported by a study done by Wang and colleagues [6] reporting association of nuclear expression with aggressive BC. ROC-1 mediated ubiquitination and degradation of nuclear proteins may contribute to bladder cancer development and progression is speculated. When ROC-1 is overexpressed specially in the nucleus skp2 of SCF complex recognizes and promotes degradation of p21 or p27 proteins and in turn causes cell unregulated proliferation, a primary alteration in cancer cells [6].

As for the P21, it displayed over expression in 83.8% of bladder cancer cases which agrees with several studies [28–30]. P21 overexpression reflected poor prognostic impact in BC which is proved by its role in tumorgenesis and cancer progression [29–33]. It has been proposed that progressive cancers accumulate p21 resulting in proliferation of the tumor cells. Also, in BC p21 can act in p53 dependent manner which is one of the predominant components of BC development or by other p53-independent pathways, including transforming growth factor beta (TGF-β) signaling [30].

High apoptotic association with P21over expression, is in concordance with Wang et al. [34], for P21 is a powerful CDK inhibitor and its protein has a cyclin binding motif and inhibits Cyclin-CDK complexes Furthermore, Cyclin A2 plays a decisive role in regulation of S phase and its deficiency will make cells accumulation in G0/G1 and decreases entry into S phase, thereby induce proliferation arrest and apoptosis [34, 35].

In the current study, P21 was expressed mainly in the cytoplasm with few cases nucleo-cytoplasmic. This is consistent with other studies which reported cytoplasmic expression of P21 in hepatocellular carcinoma and BC respectively [33, 35]. Receptor tyrosine kinases and downstream components such as the Akt/PKB serine/threonine kinase mediated P21 phosphorylation are essential for P21 localization in the cytoplasm. Inhibition of Akt retained P21 in the nucleus, resulting in less P21 complex formation with CDK2. However, Akt1-dependent cytoplasmic localization of P21 occurs in a variety of cancers where it promotes tumorigenesis by inhibiting proteins essential for apoptosis [36].

Association of cytoplasmic P21with poor pathologic features (as muscle invasion, advanced pathological stage, presence of necrosis and increased mitosis) are in agreement with Wei et al. [30]. cytoplasmic P21 functions as an oncogene, therefore promoting cancer cell proliferation and progression through the cell cycle, so correlates positively with aggressive tumors and poor prognosis, whereas nuclear P21 was reported be involved in the pro-differentiating and senescence-promoting effects [37, 38].

In the present study; CAIX was expressed in only 4 out of 15 cases of normal urothelium while significantly expressed in malignant cases, compatible with other studies [39, 40]. This might be justified by the basic function of CAIX to help produce and maintain an intracellular pH (pHi) favorable for tumor cell growth and survival, while at the same time participating in the generation of an increasingly acidic extracellular space, facilitating tumor cell invasiveness [41].

In our study CAIX showed cytoplasmic and nuclear pattern of expression matched with many other studies [42–44]. It might be explained as; alternative splicing of the CAIX transcript, besides the predominant, full-length CAIX mRNA, an alternatively spliced variant has been detected and is constitutively expressed at very low levels and codes for a truncated, cytoplasmic/secreted form of CAIX without enzymatic activity. This is very important for studies related to hypoxia-related expression of CAIX, where the presence of the alternatively spliced variant is hypoxia-independent and can provide a false-positive signal [45].

Association of CAIX overexpression with poor pathologic features as high grade UC (*P* = 0.01) and desmoplastic stroma (*P* = 0.024), were reported by others [13, 46]**,** providing further evidence of CAIX as an aggressive marker in bladder cancer and a promising targeted therapy by its inhibition.

Desmoplastic stromal reaction is a strong risk factor associated with metastasis. Hypoxia plays a role by promoting neo-angiogenesis via growth factors secreted by tumor cells and may lead to remodeling of tumor stroma and cause hypoxia inducible metastasis [47]. It is also associated with tumor necrosis regulating secretion of many factors involved in stroma formation as fibronectin, collagens, and metalloproteases [48]. Others propose that hypoxia could induce Mesenchymal - Epithelial transition, moreover, changes in intracellular and extracellular pH could be responsible for this kind of stromal reaction [46, 49, 50]. Hypoxic markers enhance glycolytic metabolism and lead to the generation of excess lactate, in this context, acidic pH is known to contribute to tumor cell invasiveness and could be an important contributing factor for the poor prognosis [50].

In this study, all the female cases showed significant expression of CAIX (*P* = 0.02). Many women ignore the most basic symptom (blood in the urine) which they may associate with menstruation or menopause. As a result, a bladder cancer diagnosis can be overlooked for a year or more [51]. Our results are supported by other studies [52].

Tumor necrosis is believed to represent the endpoint of severe, chronic hypoxia distal to functional blood vessels. CAIX expression related to necrosis and its predominance at the periphery of necrotic tumor tissue, indicates that CAIX positive tumor cells are more resistant to hypoxia and possess a growth advantage and determine its role in the protection of tumor cells from hypoxia and acidosis [43, 53]. Many studies reported a significant association between CAIX expression and presence of necrosis in different tumor types [46, 54, 55].

In the current study, although 70% of UC that displayed necrosis (14/20) expressed CAIX, yet there was no significant association. The reasons for the discrepancies with other studies are unknown; it might be related to the small sample size included in the current study.

However, evaluation of CAIX in the peri-necrotic areas and away from the necrotic areas revealed that all cases that expressed CAIX in the peri-necrotic areas were negative in the areas away from necrosis (*P* = 0.014), so the expression is increased around necrosis. This result hypothesizes that the necrosis may represent regions where CAIX expression has maintained a high level of proliferation through the maintenance of a more uniformly alkaline intracellular pH. This would increase metabolic demand and deplete substrates in the hypoxic milieu. Also, necrosis induces infiltration of macrophages which may contribute to an aggressive tumor phenotype and necrosis is associated with poor prognosis in many cancer types. So the well-recognized observation of CAIX staining around necrotic areas may reflect a role in necrosis [54].

In our study, there was a significant association between CAIX expression and presence of bilharziasis (*P* = 0.046). This might be explained as; chronic inflammation induced by bilharzial infection, had been identified as a key inducer of epithelial mesenchymal transition (EMT) during cancer progression through upregulation of mesenchymal markers and thus promoting metastasis [56, 57]. CAIX considered an EMT inducer [58] and a mesenchymal marker [59] as it played a specific role in EMT by causing extracellular acidification which closely associated with breakdown of the extracellular matrix (ECM), growth factors and protease activation. Also, expression of CAIX leads to its co-localization with β-catenin and disruption of cell-cell adhesion. Its role in EMT keeping with its ability to sense hypoxia as several components of the tumor microenvironment have been described to elicit or enhance the EMT of cancer cells, including intratumoral hypoxia [60].

Regarding the relation between the studied markers (ROC-1, CAIX and p21) with each other, a significant inverse relationship between H- score of ROC-1 and both CAIX and P21 in BC cases was noted. This finding might be promising in using these biomarkers as a targeted therapy in treating BC in Egyptian patients by causing Knockdown of ROC-1 by siRNA silencing which induce CRL inactivation that cause accumulation of some special CRL substrates such as P21 causing arrested bladder cancer cells at G2 phase [58]. So, P21upregulation targeting different regions of its promoters are an ideal method of gain of function manipulation to suppress BC cell growth [34]. Also, Upon ROC-1 siRNA silencing, it disassembles CRL/SCF complexes and thus inhibits CRL/SCF activity. As the result, DNA replication licensing proteins CDT1 and ORC1, two well-known CRL/SCF substrates, accumulate to trigger DNA damage response, leading to G2–M cell cycle arrest, senescence and/or apoptosis in cancer cells [59].

On the other hand inhibition of CAIX could be induced by upregulating ROC-1; this idea can be used as base for treating BC by causing CAIX inhibition because ROC-1 mediate un stabilization and degradation of hypoxic markers by ubiquitation of VHL as it is a component of VHL/Cullin 2/ Elongin BC/ ROC-1 complex [27]. There was also a significant direct relationship between CAIX and P21 H-score in the current study, suggesting an association in carcinogenesis and aggressiveness of BC.

## Conclusion

ROC-1, CAIX and P21 may serve as markers of aggressiveness, poor prognosis and prediction of tumor aggressiveness in bladder carcinoma in Egyptian patients. ROC-1 could be a promising candidate biomarkers that might serve as targeted therapy in hypoxic microenvironment of BC; for ROC-1 knockdown induces up regulation of P21, leading to apoptosis of BC cells, while up regulating ROC-1 leads to inhibition of CAIX. As for CAIX it may serve as potentially important disease biomarker for the identification of high risk and poor prognostic patients who may benefit from targeted therapy.

## Data Availability

The data that support the findings of this study are available from [third party name] but restrictions apply to the availability of these data, which were used under license for the current study, and so are not publicly available. Data are however available from the authors upon reasonable request and with permission of [third party name].

## Abbreviations

ROC-1: Regulator of Cullins-1
BC: Bladder cancer
CRL: Cullin-RING ligases
CKIs: Cyclin dependent kinase inhibitors
CDKs: Cyclin- Dependent Kinases
IHC: Immunohistochemical
RBX1: RING box protein-1
CIP/KIP: CDK Interacting Protein/Kinase Inhibitor Protein
AJCC-UICC: American Joint Committee on Cancer-Union International Center Cancer staging system
NMIBC: Non-muscle- invasive bladder cancer
MIBC: Muscle invasive bladder cancer
UC: Urothelial carcinoma
TGF-β-: Transforming growth factor beta
EMT: Epithelial mesenchymal transition
